# Supplementary material for: Splice-Junction-Based Mapping of Alternative Isoforms in the Human Proteome
Source: Cell Rep. Author manuscript; Available in PMC 2020 Jan 15. (PMC6961840; doi:10.1016/j.celrep.2019.11.026)
Supplement: 2 [file NIHMS1546469-supplement-2.zip › DF1/PXD010154/Esophagus/Esophagus_35_XRCC6_NLEALALDLMEPEQAVDLTYNEGSGSK.pdf]

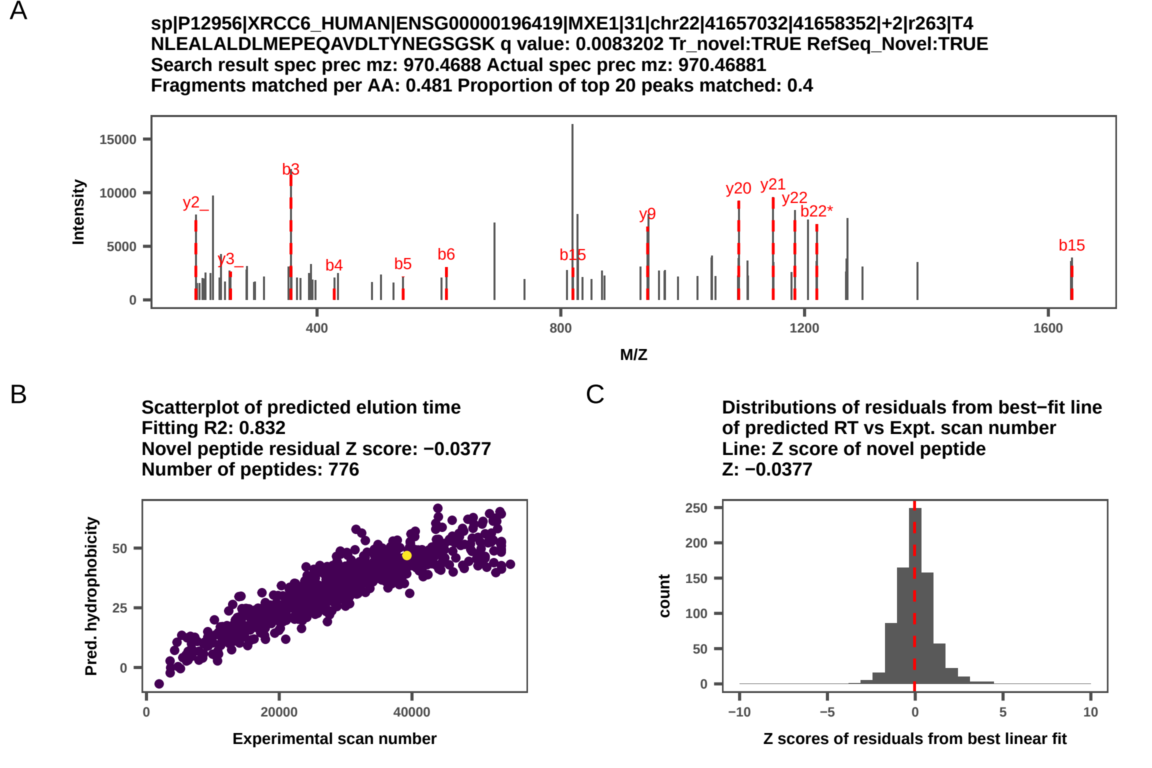
A
sp|P12956|XRCC6_HUMAN|ENSG00000196419|MXE1|31|chr22|41657032|41658352|+2|r263|T4
NLEALALDLMEPEQAVDLTYNEGSGSK q value: 0.0083202 Tr_novel:TRUE RefSeq_Novel:TRUE
Search result spec prec mz: 970.4688 Actual spec prec mz: 970.46881
Fragments matched per AA: 0.481 Proportion of top 20 peaks matched: 0.4
Intensity
15000
10000
5000
0
y2_
y3_
b3
b4
b5
b6
b15
y9
y20
y21
y22
b22*
b15
400
800
1200
1600
M/Z
B
Scatterplot of predicted elution time
Fitting R2: 0.832
Novel peptide residual Z score: −0.0377
Number of peptides: 776
Pred. hydrophobicity
50
25
0
0
20000
40000
Experimental scan number
C
Distributions of residuals from best−fit line
of predicted RT vs Expt. scan number
Line: Z score of novel peptide
Z: −0.0377
count
250
200
150
100
50
0
−10
−5
0
5
10
Z scores of residuals from best linear fit
